# Administration costs of intravenous biologic drugs for rheumatoid arthritis

**DOI:** 10.1186/2193-1801-2-531

**Published:** 2013-10-17

**Authors:** Erkki J Soini, Miina Leussu, Taru Hallinen

**Affiliations:** ESiOR Ltd, Tulliportinkatu 2 LT4, 70100 Kuopio, Finland; ESiOR Oy, Kuopio, Finland

**Keywords:** Abatacept, Biological drugs, Costs and cost analysis, Finland, Infliximab, Multivariate methods, Rituximab, Tariff, Tocilizumab, Unit cost

## Abstract

**Background:**

Cost-effectiveness studies explicitly reporting infusion times, drug-specific administration costs for infusions or real-payer intravenous drug cost are few in number. Yet, administration costs for infusions are needed in the health economic evaluations assessing intravenously-administered drugs.

**Objectives:**

To estimate the drug-specific administration and total cost of biologic intravenous rheumatoid arthritis (RA) drugs in the adult population and to compare the obtained costs with published cost estimates.

**Methods:**

Cost price data for the infusions and drugs were systematically collected from the 2011 Finnish price lists. All Finnish hospitals with available price lists were included. Drug administration and total costs (administration cost + drug price) per infusion were analysed separately from the public health care payer’s perspective. Further adjustments for drug brand, dose, and hospital type were done using regression methods in order to improve the comparability between drugs. Annual expected drug administration and total costs were estimated. A literature search not limited to RA was performed to obtain the per infusion administration cost estimates used in publications. The published costs were converted to Finnish values using base-year purchasing power parities and indexing to the year 2011.

**Results:**

Information from 19 (95%) health districts was obtained (107 analysable prices out of 176 observations). The average drug administration cost for infliximab, rituximab, abatacept, and tocilizumab infusion in RA were €355.91; €561.21; €334.00; and €293.96, respectively. The regression-adjusted (dose, hospital type; using semi-log ordinary least squares) mean administration costs for infliximab and rituximab infusions in RA were €289.12 (95% CI €222.61–375.48) and €542.28 (95% CI €307.23–957.09). The respective expected annual drug administration costs were €2312.96 for infliximab during the first year, €1879.28 for infliximab during the forthcoming years, and €1843.75 for rituximab. The obtained average administration costs per infusion were higher (1.8–3.3 times depending on the drug) than the previously published purchasing power adjusted and indexed average administration costs for infusions in RA.

**Conclusions:**

The administration costs of RA infusions vary between drugs, and more effort should be made to find realistic drug-specific estimates for cost-effectiveness evaluations. The frequent assumption of intravenous drug administration costs equalling outpatient visit cost can underestimate the costs.

## Background

Finland has a decentralized and mostly taxation-funded public health care system (e.g. Teperi et al. 2009; OECD 2005; Häkkinen 2005; Hermanson et al. 1994). The responsibility of health care organization resides with municipalities which are required by law to belong to consortiums (i.e. joint municipal boards) that organize specialized health care services for their residents. Consortiums govern health care districts (20 mainland main districts in total; Åland Islands excluded) that are funded by the municipalities that belong to the consortium. Essentially, the patients’ home municipalities pay the intravenous (IV) drug administration and drug costs in this system. Because the health care districts are regarded as non-profit organizations and the invoicing of municipalities is based on cost prices (“absorption costing”), the price lists of health care district hospitals can be used to estimate the infusion costs for the Finnish public payer.

The publications explicitly reporting infusion times, drug-specific administration costs for infusions or real-payer IV drug cost are few in number. Yet, administration costs are needed in the health economic evaluations assessing intravenously-administered drugs. In this study the primary objective was to estimate the pooled and drug- and dose-stratified average as well as adjusted mean administration costs of biologic IV drugs that are given as infusions at Finnish hospitals to treat rheumatoid arthritis (RA) in adults. The obtained estimates are compared with the purchasing power adjusted costs used in published health economic assessments.

## Methods

The biologic IV drugs included in the analysis were infliximab (IFX), rituximab (RTX), abatacept (ABA) and tocilizumab (TCZ). ABA (750 mg for patient weighting 60–100 kg) and TCZ (8 mg/kg) are indicated for RA, IFX is indicated for RA (3 mg/kg), Crohn’s disease (5 mg/kg) and psoriasis (3–5 mg/kg), and RTX is indicated for RA (500–1000 mg), chronic lymphocytic leukaemia (375–500 mg/m^2^) and non-Hodgkin’s (follicular) lymphoma (375 mg/m^2^). Based on the summaries of product characteristics and Finnish RA practice, IFX is given as 2–3 hour, RTX is given as 3.5–5 hour, TCZ is given as a 1.0–1.5 hour and ABA is given as a thirty minute infusion (e.g. Soini et al. 2012a).

### Data collection

A systematic collection of the price lists of Finnish health care districts and public and private hospitals was performed. The health care districts invoice the municipalities on the basis of these price lists and as such the reported prices represent the 'true’ cost for the public payer. At first, cost price lists for 2011 were sought from the service providers’ internet pages. If the price list was not published, a standardized e-mail enquiry was sent to the service providers (two reminders were sent). All possible hospitals (i.e. university, central district, district and private hospitals) potentially giving biologic drug infusions for RA were included to the enquiry.

The IV treatment cost prices for RA were gathered from the price lists through a keyword search of electric files and manual inspection if no matches were obtained with the keyword search. The following key words were used: drug brand names (Remicade, Mabthera, Rituxan, Orencia, RoActemra, Actemra), the name of the active substance in Finnish (infliksimabi, rituksimabi, abatasepti, tosilitsumabi) or in English (infliximab, rituximab, abatacept, tocilizumab). In the manual inspection, all observations which could be clearly interpreted as a particular IV drug were included (including “expensive / biologic drug infusions to treat RA”). The following data were extracted: service provider, hospital type, medical speciality, procedure code, drug name, dosing, infusion list cost price, items included in the list cost price (drug alone, administration alone or both drug and administration) and source.

The final data set was obtained by excluding observations that did not match the study objective. The infusions given by clearly unrelated medical specialities (paediatrics, neurologic, haematology, lung diseases, gynaecology, ear, nose and throat diseases, oncology, surgery and skin diseases) were excluded because of the dosing variation in different diseases and/or because these list cost prices included other drugs outside the scope of this analysis.

In the cases, when it was impossible to separate the drugs or no specific price for particular drug was given, “expensive RA drugs” class was used for them. Expensive RA drugs class include a variety of RA drugs which were not specified in sufficient details in the price lists, and mostly infliximab, abatacept and rituximab infusions were involved. This class was included to have all the possible cost data available for the regression models.

### Cost classification

The following cost categories were analysed separately based on the list cost prices

1. The administration costs (the main outcome of the analysis) per infusion: as reported in the price list, or alternatively the dose-dependent wholesale price of the drug was subtracted from the total costs, if the drug administration price was not reported separately from the drug price. The subtraction was performed only when the drug name and the dose information were presented, and was therefore not done for the expensive RA drugs class.
2. The total costs per infusion: as reported in the cost price lists.

The obtained list prices were also classified into RA-indicated adult doses (based on summaries of product characteristics and, if needed, assumed weight of 73 kg (Paturi et al. 2008; Soini et al. 2012a)) and atypical doses (higher or lower). The RA-indicated doses for adults in this analysis were 3 mg/kg for IFX, 500–1000 mg for RTX, 750 mg for ABA and 8 mg/kg for TCZ. A further subgroup included a pool of biologic antirheumatic drugs (bDMARD) with or without dosing information and for which the drug brands were not explicitly stated in the price lists.

### Statistical analysis

Unadjusted pooled averages and average values stratified for drug doses and drug brands were estimated for administration and total costs. The cost differences between the intravenous biologics were assessed with a non-parametric test (Wilcoxon signed rank test) due to a modest number of observations in subgroups (p-value below 0.05 was considered to indicate statistical significance).

Regression analyses were performed to obtain adjusted drug administration and total cost estimates and to allow multivariate statistical comparisons between the drugs. The costs were adjusted for drug doses (dummies for low and high doses), drug brands (dummies for ABA, TCZ and RTX) and hospital type (dummies for university hospital and regional hospital); the contrast was IFX with RA-indicated dose in central hospital. Regression analyses were performed using ordinary least squares (OLS), OLS with logarithmic (ln) conversion for the cost variable using Duan’s (Duan 1983) smearing (LN-OLS) and generalized linear modelling (GLM) (see e.g. Hallinen et al. 2006; Soini et al. 2012b) with the fittest distribution and link function. Akaike information criteria (AIC), Bayesian information criteria (BIC), log likelihood (LL) and coefficient of determination (R^2^) were reported.

The best regression model for heterogeneity adjustment between drug brands (“significant” model) was determined using a procedure based on AIC and R^2^: the least significant coefficients were excluded from the model until AIC did not improve anymore and R^2^ did not drop. The best model type (OLS, LN-OLS or GLM) among adjustment models was determined using AIC and BIC. Analyses were implemented in Stata v10.

### Administration costs in previous publications

A rapid review was done of the existing publications (15^th^ August 2011) from the PubMed and Cochrane databases, and from potential Finnish medical, pharmacist and social science scientific journals in order to find out the published administration costs for IV biologics (IFX, RTX, ABA, TCZ) in various diseases and to reflect the RA administration costs of this study. The search terms included (in English and Finnish): cost, price, pricing, resource, resourcing, budget, economic OR burden AND Remicade, Rituxan, Mabthera, Orencia, Actemra, RoActemra, infliximab, rituximab, abatacept, atlizumab OR tocilizumab. Total costs were not searched, because the drug prices during the base year in the given country were unknown, and the pricing as well as reimbursement (payment system) of the drug costs may differ significantly due to local setups (see e.g. Hallinen and Soini 2011). Publications were included in the review if the used currency, the base costing year, the drug brand and the given drug administration cost were clearly stated.

In order to obtain comparability with the Finnish cost prices for 2011 and average costs estimated in the main analysis, the costs obtained from the literature review were converted to their base year Finnish costs using purchasing power parities (PPP) obtained from OECD and were presented in 2011 values using the official Finnish health care price index obtained from Statistics Finland.

## Results

The cost price data for 2011 from 19 (95%) health districts were obtained for the raw data. No private hospitals sent their price data and two private hospitals stated that they do not administer these infusions. As a result, the costs presented here capture the public health care payer’s perspective. The obtained sources of the cost price data are presented in the Appendix 1.

The raw data consisted of 176 list cost prices in total. After exclusion of irrelevant cost prices (i.e. list prices not matching the study objective), 107 cost prices (60.8% of raw data) remained for inclusion in the analysis. The cost prices from RA departments (four prices, 3.7%), internal disease departments treating RA (63 prices, 58.9%) and of expensive RA drugs (forty prices, 37.4%) were included. The costs of biologic RA drug infusions varied between different Finnish public service providers.

### Pooled costs

For crude administration cost analysis and total costs analysis, 82 (76.6%) and 106 valid list prices (99.1%), respectively, were included in the sample. The pooled (i.e. all drug brands and doses combined) crude average administration costs were €404.06 (S.D. €269.04, n=82) and total costs were €2826.28 (S.D. €1601.23, n=106) per infusion.

The pooled average administration costs for doses indicated for adult RA were €400.36 (S.D. €259.61, n=63) and total costs were €2831.72 (S.D. €1690.41, n=63). The pooled average administration costs and total costs were €416.32 (S.D. €305.55, n=19) and €2701.65 (S.D. €827.15, n=23), respectively, for doses deviating from adult RA indication. For twenty list prices (total costs average €2955.35, S.D. €2031.26) the dosing was not stated. There were no statistically significant differences between the costs matching the adult RA indication and undefined doses or doses outside the indicated doses.

The summary statistics according to drug brand regardless of used doses are represented in Table 1. The average administration costs were highest for RTX and lowest for IFX, while the average total costs were highest for IFX and lowest for ABA. The difference between administration costs as well as total costs of the most and least expensive drug were statistically significant (p<0.01 for both cost types in all analyses).

**Table 1:** **Total and administration costs (€2011) of public health care payer by drug regardless of dosing**

Drug	Administration (€, N 82) per infusion				Total costs^a^(€, N 106) per infusion			
	***N***	***Average***	***S.D.***	***Range***	***N***	***Average***	***S.D.***	***Range***
Infliximab	52	330.87^b^	158.27	25–728	51	3128.21^c^	1560.96	647-6648
Rituximab	20	578.38^b^	425.65	15–1572	20	2612.37^c^	1412.06	342-6147
Abatacept	5	455.60	138.22	334–688	5	1422.80	440.05	1120-2176
Tocilizumab	5	414.63	202.00	263–728	5	1454.00	416.33	1204-2176
Expensive infusion with RA dose	0	NR	NR	NR	5	2873.20	1056.19	1695-3895
Other expensive infusion	0	NR	NR	NR	20	2955.35	2031.26	1390-10213

RA = rheumatoid arthritis. N = number of observations. S.D. = standard deviation. NR = not reported. ^a^ Includes administration and drug costs. ^b^ Average IFX administration cost was statistically significantly different from average RTX administration cost (p-value = 0.007). ^c^ Average IFX total cost was not statistically significantly different from average RTX total cost (p-value = 0.175).

### The average costs stratified by dose and drug brand

The stratified results by drug using the indicated adult RA dose or other dose are represented in Table 2. The average administration cost with the indicated adult RA dose was €355.91 for IFX, €561.21 for RTX, €334.00 for ABA and €293.96 for TCZ. The average total costs for adult RA doses were €2243.10 for IFX, €3619.17 for RTX, €1450.00 for ABA and €2176.00 for TCZ. RTX 500–1000 mg administration was not significantly more costly compared with IFX 3 mg/kg administration whereas RTX 500–1000 mg total costs were significantly higher compared with IFX 3 mg/kg total costs (p<0.01).

**Table 2:** **Administration and total costs (€2011) by the indicated common and non-indicated adult RA dose**

Drug	RA dose	Administration (€, N 82) per infusion				Total costs^a^(€, N 106) per infusion			
		***N***	***Average***	***S.D.***	***Range***	***N***	***Average***	***S.D.***	***Range***
Infliximab	Yes	11^bc^	355.91	125.45	123–543	10^ef^	2243.10	110.92	1990–2410
	No	41^b^	324.15	166.69	25–728	41^e^	3344.09	1673.13	647–6648
Rituximab	Yes	6^cd^	561.21	516.78	142–1572	6^fg^	3619.17	516.78	3200–4630
	No	14^d^	586.37	402.34	15–1560	14^g^	2180.88	1463.94	342–6147
Abatacept	Yes	1	334.00	NS	NS	1	1450.00	NS	NS
	No	4	486.00	138.97	376–688	4	1416.00	507.83	1120–2176
Tocilizumab	Yes	1	293.96	NS	NS	1	2176.00	NS	NS
	No	4	444.80	219.86	263–728	4	1273.50	117.91	1204–1450

RA = rheumatoid arthritis. N = number of observations. S.D. = standard deviation. NS = not stated. ^a^ Includes administration and drug costs. ^b^ IFX using RA dose did not have significantly lower administration costs compared with IFX not using RA dose. ^c^ RTX with RA dose was not significantly more costly compared with IFX with RA dose. ^d^ RTX using RA dose did not have significantly lower administration costs compared with RTX patients using RA dose. ^e^ IFX patients using RA dose had significantly lower total costs compared with IFX patients not using RA dose (p-value = 0.013). ^f^ RTX patients using RA dose had significantly lower total costs compared with RTX patients not using RA dose (p-value = 0.011). ^g^ RTX with RA dose was significantly more costly compared with IFX with RA dose (p-value = 0.001).

The variability in costs was reduced significantly, when the commonly indicated and non-indicated RA drug-specific doses were taken into account. The cost variability was lower with the indicated RA dose when compared to the variability of costs based on the non-indicated dose. The average administration costs per infusion with the non-indicated adult RA doses were lowest for IFX and highest for RTX. The average total costs per infusion with the non-indicated adult RA doses were lowest for TCZ and highest for IFX. IFX and RTX with the indicated adult RA dose were not significantly different from their non-indicated adult RA dose whereas IFX and RTX with the indicated adult RA dose were significantly less costly compared with their non-indicated adult RA dose (p<0.05 for both in all analyses).

### Adjusted mean costs

The models used for drug administration and total cost adjustments (drug brand, dose, hospital type) and multivariate comparisons are represented in Tables 3 and 4, respectively. Dosing was separated for low, RA and high drug doses in the regression models. Robust estimation was used in all models.

**Table 3:** **Statistical models for adjusted per-infusion administration costs (€2011)**

Parameter^a^	OLS (full)	OLS (sign.)	LN-OLS (full)	LN-OLS (sign.)	GLM (full)^b^	GLM (sign.)^b^
	***Coef. 95% CI***	***Coef. 95% CI***	***Coef. 95% CI***	***Coef. 95% CI***	***Coef. 95% CI***	***Coef. 95% CI***
University hospital	-133.07	-139.23*	-0.5129	-0.6008*	-134.57**	-172.52***
	-272.85–6.71	-252.98–-25.49	-1.1299–0.1041	-1.1449–-0.0567	-232.93–-36.21	-245.95–-99.09
District hospital^c^	10.29	NR	0.1467	NR	63.02	NR
	-130.59–151.17		-0.2432–0.5366		-57.96–184.01	
Rituximab	332.89**	332.33**	0.6370*	0.6290*	327.67***	318.99***
	94.04–571.73	96.44–568.21	0.0591–1.2148	0.0608–1.1971	151.78–503.57	144.90–493.08
Abatacept^d^	191.62*	186.47**	0.7960*	0.7225*	262.52***	245.90***
	34.78–348.47	47.20–325.75	0.1767–1.4152	0.1431–1.3019	171.59–353.45	150.93–340.87
Tocilizumab^d^	195.76	192.98	0.7995*	0.7598*	233.88***	230.25***
	-57.66–449.19	-51.25–437.20	0.0938–1.5051	0.0438–1.4758	116.70–351.07	102.52–357.99
Low dose	-88.12	-86.51	-0.4786	-0.4556	-153.19***	-146.56***
	-255.55–79.30	-251.47–78.45	-0.9849–0.0277	-0.9427–0.0314	-228.44–-77.94	-221.47–-71.66
High dose	147.71*	150.86*	0.4219*	0.4669**	101.20*	110.66**
	8.72–286.69	9.02–292.70	0.1041–0.7396	0.1804–0.7534	20.06–182.34	31.94–189.38
Constant	313.92***	318.71***	5.5986***	5.6668***	330.90***	363.50***
	196.40–431.44	230.55–406.86	5.2776–5.9197	5.4054–5.9282	243.30–418.50	293.01–433.99
*Tests*						
N	82	82	82	82	82	82
R^2^	0.29	0.29	0.29	0.28	NR	NR
Prob	0.015*	0.008**	0.002**	0.002**	NR	NR
LL	-560.38	-560.39	-86.94	-87.23	-567.96	-568.19
AIC	1136.75	1134.78	189.87	188.46	1151.92	1150.38
BIC	1156.00	1151.63	209.12	205.30	1171.18	1167.23

OLS = ordinary least squares regression model. Sign. = significant model. LN-OLS = OLS regression model with logarithmic transformation in dependent parameter. GLM = generalized linear model regression. Coef. = coefficient. 95%CI = 95% confidence interval. * p-value < 0.050. ** p-value < 0.010. *** p-value < 0.001. NR = not reported. N = number of observations. R^2^ = coefficient of determination. Prob = model p-value. LL = log likelihood. AIC = Akaike information criteria. BIC = Bayesian information criteria. ^a^ Contrast is central district hospital, infliximab and indicated common adult rheumatoid arthritis dose. ^b^ Gamma distribution and linear link function in GLM provided the best fit among GLMs. ^c^ Few costs were obtained from (non-university or non-central) district hospitals. ^d^ Potentially biased upwards due to low number of observations.

**Table 4:** **Statistical models for adjusted per-infusion total (administration and drug) costs (€2011)**

Parameter^a^	OLS (full)	OLS (sign.)	LN-OLS (full)	LN-OLS (sign.)	GLM (full)^b^	GLM (sign.)^b^
	***Coef. 95% CI***	***Coef. 95% CI***	***Coef. 95% CI***	***Coef. 95% CI***	***Coef. 95% CI***	***Coef. 95% CI***
University hospital	5.35	NR	0.0243	NR	0.0100	NR
	-537.73–548.42		-0.2525–0.3011		-0.1683–0.1882	
District hospital^c^	155.44	NR	0.1171	NR	0.0614	NR
	-277.97–588.84		-0.0767–0.3109		-0.0774–0.2003	
Rituximab	807.61***	773.22***	0.2943*	0.2815*	0.3502***	0.3340***
	434.58–1180.64	398.34–1148.10	0.0545–0.5339	0.0492–0.5139	0.1974–0.5030	0.1834–0.4847
Abatacept^d^	-619.85	-713.33	-0.1826	-0.2440	-0.2158	-0.2553
	-1836.24–596.54	-1920.71–494.05	-0.6012–0.2360	-0.6516–0.1635	-0.5026–0.0710	-0.5337–0.0231
Tocilizumab^d^	70.29	9.43	0.0897	0.0548	0.0248	-0.0034
	-332.87–473.44	-395.93–414.79	-0.1444–0.3238	-0.1630–0.2715	-0.1447–0.1942	-0.1622–0.1553
Pool of bDMARDs	359.77	NR	0.1167	NR	0.1603	NR
	-747.32–1466.86		-0.2955–0.5290		-0.1443–0.4650	
Low dose	-1449.67***	-1477.90***	-0.7999***	-0.8005***	-0.7217***	-0.7325***
	-1776.25–-1123.09	-1799.91–-1155.89	-1.0147–-0.5852	-1.0041–-0.5968	-0.8763–-0.5671	-0.8823–-0.5828
High dose	2000.45***	1979.89***	0.5459***	0.5586***	0.5873***	0.5762***
	1415.46–2585.45	1453.46–2506.33	0.3814–0.7104	0.4225–0.6947	0.4546–0.7200	0.4546–0.6979
Constant	2511.29***	2626.89***	7.7763***	7.8403***	7.7980***	7.8491***
	2232.45–2790.13	2416.33–2837.45	7.6524–7.9000	7.7534–7.9273	7.7070–7.8891	7.7716–7.9266
*Tests*						
N	86	86	86	86	86	86
R^2^	0.76	0.76	0.71	0.71	NR	NR
Prob	0.00***	0.00***	0.00***	0.00***	NR	NR
LL	-688.32	-689.07	-25.83	-26.91	-758.35	-758.42
AIC	1394.65	1390.14	69.66	65.81	1534.70	1528.84
BIC	1416.74	1404.86	91.75	80.54	1556.78	1543.56

OLS = ordinary least squares regression model. Sign. = significant model. LN-OLS = OLS regression model with logarithmic transformation in dependent parameter. GLM = generalized linear model regression. Coef. = coefficient. 95%CI = 95% confidence interval. * p-value < 0.050. ** p-value < 0.010. *** p-value < 0.001. NR = not reported. bDMARD = biologic antirheumatic drug. N = number of observations. R^2^ = coefficient of determination. Prob = model p-value. LL = log likelihood. AIC = Akaike information criteria. BIC = Bayesian information criteria. ^a^ Contrast is central district hospital, infliximab and indicated common adult rheumatoid arthritis dose. ^b^ Gamma distribution and log link function in GLM provided the best fit among GLMs. ^c^ Few costs were obtained from (non-university or non-central) district hospitals. ^d^ Can be biased upwards due to low number of observations.

Based on the best-fitting model (LN-OLS, semi-log OLS, logarithmic distribution assumption for administration costs; Table 3), the adjusted administration mean costs with the indicated RA dose were €289.12 (95% CI €222.61–375.48) for IFX and €542.28 (95% CI €307.23–957.09) for RTX per infusion in a central hospital. ABA and TCZ administration costs as well as regional hospital administrations were based on a few relatively high cost estimates and were, thus, potentially biased upwards in the regressions. The adjusted administration costs for higher doses were significantly more costly (p<0.01) in comparison with the administration of indicated adult RA doses, whereas there was no statistically significant difference between the adjusted administration costs for lower doses and indicated adult RA doses.

Based on the best-fitting LN-OLS model, the adjusted total mean costs (Table 4) with the indicated RA dose were €2540.97 (95% CI €2329.48–2771.93) for IFX and €3367.08 (95% CI €2669.11–4247.99) for RTX per infusion. ABA and TCZ total costs were based on only a few cost estimates and may not be representative. RTX total costs were significantly higher (p<0.05) in comparison with IFX, when adjusted for drug, dose and hospital type. The total costs of TCZ, ABA or pool of bDMARDs did not differ statistically significantly from the total costs of IFX, when adjusted for dose and hospital type. High doses were significantly more costly and low doses significantly less costly in comparison with the indicated adult RA doses (p<0.001 for both).

Based on these adjusted costs per infusion and the summaries of product characteristics for the annual number of infusions, the public payer average annual administration / total costs directly related to the respective biologic RA drug infusions are estimated to be €2312.96 / €20,327.76 for IFX during the first year (eight infusions), €1879.28 / €16,516.31 for IFX during the forthcoming years (6.5 infusions) and €1843.75 / €11,448.07 for RTX (2×1.7 infusions Soini et al. 2012a), per year.

### Reference: published costs

In the review of published literature, 56 articles reporting 76 costs were found for further review. Valid cost data for infusion administration was reported in 20 articles (Beresniak et al. 2011; Bodger et al. 2009; Chen et al. 2006; Cummins et al. 2011; Dretzke et al. 2011; Fonia et al. 2010; Hallinen et al. 2010; Jobanputra et al. 2002; Kasteng et al. 2008; Lekander et al. 2010; Lindsay et al. 2008; Lyseng-Williamson and Foster 2004; Malottki et al. 2011; Punekar et al. 2010; Rodgers et al. 2011; Soini et al. 2011; Tsai et al. 2008; Vera-Llonch et al. 2008a, b; Woolacott et al. 2006; Yuan et al. 2010) with 28 costs. In many published cost-effectiveness evaluations, the IV drug administration cost was based on assumption (e.g. cost equalling outpatient or nurse visit).

In the literature review of the existing publications including IFX, RTX, ABA or TCZ and administration costs, the pooled average administration cost regardless of drug, dose and disease was €199.60 (range €76.87–484.57, S.D. €94.92, n=28). The average administration cost was €158.75 (range €76.87–212.79, S.D. €45.44, n=6) with ABA, €169.67 (range 76.87–230.97, S.D. €59.14, n=6) with RTX and €226.15 (range 76.87–484.57, S.D. €109.62, n=16) with IFX (disease and dose ignored). The average administration cost was €163.08 (n=1) with ulcerative colitis, €176.35 (range €76.87–270.23, S.D. €54.30, n=17) with RA, €165.02 (range €99.06–230.97, S.D. €65.96, n=2) with non-Hodgkin’s (follicular) lymphoma, €247.88 (range €108.02–484.57, S.D. 141.98, n=4) with psoriatic arthritis and psoriasis, and €276.58 (range €163.08–484.57, S.D. €129.31, n=4) with Crohn’s disease (drug ignored).

The average administration costs stratified by drug and disease obtained from the published literature are presented in Table 5. The lowest (€158.75) RA average administration cost was with ABA and the highest (€193.92) with IFX. The costs assumed in the publications were generally lower than the estimates based on our sample (1.8–3.3 times lower depending on the drug and in comparison with the stratified average costs).

**Table 5:** Results of the literature review: the average administration costs (€2011)*

Drug^a^	Disease	N	Administration cost (€)	Range (€)	S.D. (€)
IFX	Rheumatoid arthritis	7	193.92	76.87–270.23	55.33
RTX	Rheumatoid arthritis	4	172.00	76.87–212.79	55.26
ABA	Rheumatoid arthritis	6	158.75	76.87–212.79	45.44
IFX	Crohn’s disease	4	276.58	163.08–484.57	129.31
IFX	Psoriatic arthritis, psoriasis	4	247.88	108.02–484.57	141.98
IFX	Ulcerative colitis	1	163.08	163.08–163.08	NS
RTX	Follicular (non-Hodgkin’s) lymphoma	2	165.02	99.06–230.97	65.96

* Costs from previous article publications were converted to Finnish purchasing power and reported in year 2011 value. N = number of observations. S.D. = standard deviation. IFX = infliximab. RTX = rituximab. ABA = abatacept. NS = not stated. ^a^ No administration costs for tocilizumab were found.

## Discussion

The administration and total costs of biologic drug infusions for RA vary between different Finnish public service providers based on the cost price information for 2011. Based on the drug and dose stratified analysis, the average infusion administration / total costs with the common indicated RA adult doses were €355.91 / €2243.10 for IFX, €561.21 / €3619.17 for RTX, €334.00 / €1450.00 for ABA and €293.96 / €2176.00 for TCZ, respectively, per infusion.

The dose and hospital type adjusted administration / total mean costs with RA dose were €289.12 / €2540.97 for IFX and €542.28 / €3367.08 for RTX based on the best-fitting significant (LN-OLS) model, respectively, per infusion. The respective annual administration / total costs were estimated to be €2312.96 / €20,327.76 for IFX during the first year, €1879.28 / €16,516.31 for IFX during the forthcoming years, and €1843.75 / €11,448.07 for RTX, per year. The rank order of the drugs in terms of their cost remained in the different adjustments made and showed the robustness of findings. Due to the few ABA and TCZ costs found, reliable adjusted means for them could not be estimated using regression or any other multivariate methods. As usual with skewed cost distributions, the normal distribution assumption (common OLS) cut the distribution tails and the suitability of GLM did not exceed the suitability of LN-OLS (see e.g. Hallinen et al. 2006; Soini et al. 2012b).

These results were based on the list price (tariff) information obtained from the Finnish public hospitals. To ascertain the coverage of the sample, we asked the prices from the Finnish private hospitals and obtained none (two said that they do not give such infusions). This was an expected finding, because patient co-payments are considerably higher in the private sector. In addition to paying more for the administration of the IV-drug in the private sector, the patients would also have to pay the price of the drugs in full since the studied IV-administered drugs are not covered by the Finnish drug reimbursement system. Instead, in the publicly funded hospitals the patient co-payment is a negligibly small proportion of the total drug and drug administration costs (maximum 27.40 euros/visit). Due to this characteristic of the Finnish multi-channelled health care financing system, IV-drug infusions are not given by (majority of) the private sector service providers.

These differences between drugs in terms of their administration costs can be a result of multiple reasons and the impact of most obvious one (number of infusions per year) was shown. We controlled the hospital type and dosing with multivariate methods. However, what was left outside of the scope of this manuscript was the actual pricing process (i.e. different providers may have different systems to price their drug administration services), which means that we assessed the unit costs for drug administration and health economic evaluation (technical and/or allocative efficiency) was beyond the scope of this manuscript. Obvious reasons for the differences in infusion administration costs include resources (labour and materials involved) and their use (productivity) as well as their unit costs (more or less costly labour, buildings and equipment) which are put together in the production process of services that may or may not be efficient.

Furthermore, the Finnish hospital tendering system (pharmaceuticals formulary) may have an impact to the costs. Meanwhile the total costs per infusion are valid as such, the administration costs per infusion are likely to be too low in comparison to the true administration cost invoiced from the municipalities due to the fact that some hospitals and districts have power to negotiate lower costs for their drug purchases (i.e. which results to situation where we subtracted too much from the total costs as the drug costs and results to lower than true administration costs). Despite of this inaccuracy in the analysis, the administration costs obtained from the lists are higher than the outpatient visit costs compared to the Finnish average (Hujanen et al. 2008) or specific lists from which the total and administration costs were analysed.

The presented pooled estimates describe the data and apply for situations where drug, dosing and/or hospital type are not taken into account. Using the stratification, we aimed to present drug- and dose-specific costs for all IV drugs, some of which may present with a low number of list prices. With the statistical modelling of cost data, we could adjust the differences (heterogeneity) in list prices between the different IV drugs with enough list prices and enable the statistical comparison of costs related to different IV drugs when they would be given in equivalent settings.

Finding the optimal time to start biologic treatment for RA patient is challenging especially when conventional therapies evolve (e.g. Fautrel 2012) and the administration cost of intravenous biologic drug may depend, for example, on the number of infusions received and other patient characteristics which we could not take into account in our analyses. In addition, there are five noteworthy facts, which need to be taken into account when interpreting and applying the results of this study.

The first and most important is the international comparability of costs. Although the relative Finnish health care cost, measured as the proportion of gross-domestic product consumed, to health care is low (around 9%), the previous Finnish publications including the cost of IV drug administration (Hallinen et al. 2010; Soini et al. [Bibr CR31][Bibr CR29]) have used higher estimates compared with the average estimates based on the publications. However, in order to improve the international comparability, the main results of this study can be converted to foreign values using PPPs. The statistically adjusted (LN-OLS) mean administration cost per IFX infusion for an adult (3 mg/kg) is, for example, AU$476.17 / CA$375.62 / FR€264.89 / DE€243.85 / JPY32,654.33 / RUB5501.69 / SEK2723.49 / UK£201.40 / US$305.53 in Australia / Canada / France / Germany / Japan / Russian Federation / Sweden / United Kingdom / United States when converted to local purchasing power and currency in 2011 using suitable PPP. The respective mean administration costs per RTX 500–1000 mg infusion are AU$893.12 / CA$704.53 / FR€496.83 / DE€457.37 / JPY61,247.21 / RUB10,319.10 / SEK5108.24 / UK£377.75 / US$573.06.

The second noteworthy fact is the perspective and content of the cost estimate. In comparison with the purchasing power adjusted and 2011 valued average values obtained from the published RA literature (IFX €193.92, RTX €172.00, ABA €158.75), our price list-based stratified averages seem high (1.8–3.3 times). This is not surprising since in many publications the cost of IV drug administration is based on “outpatient visit assumption” which may significantly underestimate the cost due to, for example, ignorance of different infusion times and severity of patient’s condition, the need for monitoring the patient during and after infusion as well as the potential need for hospital beds and associated resources. Consequently, academic price lists may overlook some cost items meanwhile they have to be included in the local price lists in order to keep the hospital working. This path leads to the perspective discussion: the local price lists may reflect the 'true’ opportunity cost (shadow price) of the service for the payer – that is what the municipality is likely to lose in terms of euros used for health care elsewhere when some resident have a specific service (see e.g. Hallinen et al. (2012); Soini (2008), for further discussion regarding the opportunity costs).

In some countries home-based administration may be done. However, home-based administration of IV biologic RA drugs rarely done in Finland. These observations result to question, how comparable and credible is the key assumption used in multiple evaluations (i.e. outpatient visit costs between nations). For example, the United Kingdom Personal Social Services Research Unit (PSSRU) unit cost list (Curtis 2011) shows the cost of £147 (€169.38; based on the average 1€ = £0.86788 during the year 2011) for the outpatient visit in year 2011 which is significantly (2.9-times) less than e.g. administration cost in the HTA submission (Woolacott et al. 2006, €484.57 in year 2011 value). Yet, the UK outpatient visit cost is more in line with the Finnish (Hujanen et al. 2008) outpatient visit cost estimate (€202.29 in 2011 value). The outpatient visit tariffs in Finnish hospital price lists are well in line with these estimates meanwhile the cost for biologic drug infusion administration can be higher. Unfortunately, unit cost list like Hujanen et al. (2008) and Curtis 2011 are not available for many countries, including Sweden, and the detailing of the unit cost lists and contents of the specific unit cost can vary considerably between the nations.

The relatively high costs of IFX and RTX in this study can be, for the minor part, explained by the fact that IFX is used as a combination treatment with methotrexate and RTX is used together with methylprednisolone (despite methotrexate and methylprednisolone being relatively affordable), whereas ABA and TCZ can be used as monotherapies or as combination therapies. More obvious explanation is the infusion time, IFX and RTX infusions take time. In the Finnish “official” price list for health care resources (Hujanen et al. 2008), the 2011 valued unit cost estimate for a public internist or oncologist outpatient visit (including tests) is €202.29 or €227.14, respectively, which are in line with the results of this study and have been used in the Finnish health economic evaluations (e.g. Hallinen et al. 2010; Soini et al. 2011). These outpatient visit costs are higher when compared with the average values of published infusion costs for RA or other diseases in the performed literature review and low compared with some of the RA-related estimates obtained in this study. Thus, “outpatient visit cost assumption” can underestimate the costs of IV drug administration in RA.

The third fact is the analytical (costing) perspective. The cost estimates in this study cover public health care payer costs only (patient co-payments excluded, maximum €27.40/visit). The level of costs estimated using the public health care price lists may be partly explained by the fact that no data from Finnish private hospitals were included in our data: in the Finnish official price list (Hujanen et al. 2008), for example, the 2011-valued unit cost for an outpatient visit to an internist was €86.28 and to a rheumatologist €78.85 (including €9.29 payment for the receptionist office; excluding tests), and for an oncologic I.V. infusion/injection taking less than two hours was €135.17 (S.D. €215.30; payments for the office excluded); all these private sector costs seem low in comparison with the public sector costs.

On the other hand, our estimates do not cover all the costs involved with IV drug administration. If we had tried to analyse the costs from the direct health care cost perspective (regardless of the payer), the analysis should have included patient co-payments (maximum €27.40/infusion) and travelling costs. The societal perspective should have also included the potential productivity losses.

The last fact is the applied indication. Based on the performed literature review, the previously published administration costs for RA differed from the administration costs for Crohn’s disease, psoriatic arthritis, ulcerative colitis or follicular lymphoma. However, the differences were due to two studies (Bodger et al. 2009; Woolacott et al. 2006) using a remarkably higher cost estimate (€484.57 according to the 2011 value) for the administration. To sum up, these facts may indicate that the drug-specific estimates provided here for RA infusion administration may be a “better than nothing” approximate estimate for diseases other than RA and further studies should be done to find the most specific unit costs for the health economic evaluations.

In order to place the results of this study in a wider context, the comparison of costs related to IV and other types of drugs needs to be done with due caution. In the case of drugs administered by health care personnel (e.g. hospital-administered IV), adherences can be known for certain. However, when the drug is taken at home, for example, the secondary non-adherence is not known (e.g. Hovstadius and Petersson 2011).

The results of our study suggest that there is uncertainty related to drug infusion costs in previously published cost-effectiveness studies. Therefore, our study highlights the need for a more careful estimation of realistic administration costs for IV-drugs in health economic evaluations. The international comparability of cost estimates and incremental cost-effectiveness results could be improved by using PPPs more frequently in the result reporting.

## Conclusions

The infusion costs vary greatly between drugs. The frequent assumption of administration costs equalling outpatient visit cost can underestimate the true costs: the average costs in this study were significantly higher than those reported in the published literature.

## Appendix 1. The list of the obtained price lists

Etelä-Karjalan sosiaali- ja terveyspiiri. Erikoissairaanhoidon hinnasto 2011.

Etelä-Pohjanmaan sairaanhoitopiiri. Palveluhinnasto 2011.

Etelä-Savon sairaanhoitopiirin kuntayhtymä. Palveluhinnasto 1.1.2011. Available: http://www.esshp.fi/downloader.asp?id=4852&type=1

Forssan seudun terveydenhuollon Ky. Hinnasto 2011. Information received by e-mail 30^th^ June 2011.

Helsingin ja Uudenmaan sairaanhoitopiiri. HUS palveluhinnasto 2011, osa 2 suoriteperusteiset sairaanhoidolliset palvelut.

Itä-Savon sairaanhoitopiirin kuntayhtymä Sosteri. Kuntalaskutuksen palveluhinnasto, erikoissairaanhoito, perusterveydenhuolto, sosiaalipalvelut.

Kainuun maakunta-kuntayhtymän esh. Palveluhinnasto 2011. Information received by e-mail 30^th^ June 2011.

Kanta-Hämeen sairaanhoitopiirin kuntayhtymä. Hinnasto 2011.

Keski-Pohjanmaan erikoissairaanhoito- ja peruspalvelukuntayhtymä Kiuru. Palveluhinnasto 2011.

Keski-Suomen sairaanhoitopiiri. Hoitopalveluiden hinnat 2011.

Lapin Sairaanhoitopiiri. Kuntalaskutuksen perusteena olevat tulosyksiköt v. 2011. Information received by e-mail 4^th^ July 2011.

Länsi-Pohjan sairaanhoitopiirin kuntayhtymä. Poliklinikkakäyntien ja hoitopäivien hintaluokat kuntalaskutuksessa 1.1.2011 lukien. Information received by e-mail 30^th^ June 2011.

Pirkanmaan sairaanhoitopiiri. Tuotteet ja hinnat 2011, 1.5.2011 alkaen. Information received by e-mail 2^nd^ August 2011.

Pohjois-Karjalan sairaanhoito- ja sosiaalipalvelujen kuntayhtymä. Tuotehinnasto 2011. Information received by e-mail 30^th^ June 2011.

Pohjois-Pohjanmaan sairaanhoitopiiri. Palveluhinnasto 2011. Available: http://www.ppshp.fi/instancedata/prime_product_julkaisu/npp/embeds/24022_Palveluhinnasto_2011.pdf

Pohjois-Savon sairaanhoitopiiri. Kliinisten erikoisalojen palvelutuotteet, suoritteet ja hinnat 2011.

Päijät-Hämeen sosiaali- ja terveyspalveluiden kuntayhtymä. Palvelut ja hinnat 2011. Available: http://kuntatoimisto.phsotey.fi/ktwebbin/ktproxy2.dll?doctype=0&docid=31363937393a31&dalid=7.12.2010%2009:18:55&extension=pdf

Raahen aluesairaalan hinnat. Information received by email 16^th^ August 2011.

Satakunnan sairaanhoitopiiri. Palveluhinnasto 2011. Available: http://www.satshp.fi/pls/wportal/url/ITEM/9759642D26A39B32E0400A0A4B0027C4

Vaasan keskussairaala. Hinnasto 2011. Information received by e-mail 30^th^ June 2011.

Varkauden aluesairaalan hinnat. Information received by e-mail 15^th^ August 2011.

Varsinais-Suomen Sairaanhoitopiiri. TYKS Suoritehinnasto 2011. Available: http://www.vsshp.fi/fi/dokumentit/28817/TYKS.pdf

Varsinais-Suomen sairaanhoitopiiri, Aluesairaalat. Suoritehinnasto 2011. Available: http://www.vsshp.fi/fi/dokumentit/28042/Aluesairaalat.pdf

Varsinais-Suomen sairaanhoitopiiri, Turunmaan sairaala liikelaitos. Hinnat 2011. Available: http://www.vsshp.fi/fi/dokumentit/26770/TMS.pdf

Varsinais-Suomen sairaanhoitopiiri. Hinnastolisäys 1–2011, 2.3.2011. Available: http://www.vsshp.fi/fi/dokumentit/27313/Lis%C3%A4ys%201-2011.pdf

Varsinais-Suomen sairaanhoitopiiri. Hinnastolisäys 2–2011, 17.5.2011. Available: http://www.vsshp.fi/fi/dokumentit/28545/Lis%C3%A4ys%202-2011.pdf

Ylä-Savon SOTE kuntayhtymä. Terveyden ja sairaanhoidon vastuualueen hinnasto 2011. Available: http://ktweb.ylasavonsote.fi/ktwebbin/ktproxy2.dll?doctype=1&docid=323031315c303231305c39373332333835342e504446&dalid=10.2.2011%2009:51:37&extension=pdf

## Abbreviations

95% CI: 95 per cent confidence interval
ABA: Abatacept
AIC: Akaike information criteria
bDMARD: Biologic antirheumatic drug
BIC: Bayesian information criteria
Coef.: Coefficient
GLM: Generalized linear modelling
IFX: Infliximab
IV: Intravenous
kg: Kilogram
LL: Log likelihood
LN-OLS: Ordinary least squares regression model with natural logarithm of dependent variable
mg: Milligram
N: Number of observations
NR: Not reported
NS: not stated
OECD: Organisation for economic cooperation and development
OLS: Ordinary least squares
PPP: Purchasing power parity
R2: Coefficient of determination
RA: Rheumatoid arthritis
RTX: Rituximab
S.D: Standard deviation
TCZ: Tocilizumab.
